# The ‘teabag method’: tick feeding protocol and the effects of tick feeding on hematological parameters in the canine host

**DOI:** 10.1186/s13071-026-07271-x

**Published:** 2026-02-10

**Authors:** Jonathan Ferm, Roman Ganta

**Affiliations:** Department of Pathobiology and Integrative Biomedical Sciences, College of Veterinary Medicine, Bond Life Sciences Center, University of Missouri, Columbia, MO USA

**Keywords:** Infestation, Tick-borne diseases, Canine host, Containment, Experimental tick feeding

## Abstract

**Background:**

Hard tick infestation occurs naturally in humans, domestic animals, and livestock species. Upon feeding, ticks transmit a wide variety of pathogens that may result in serious diseases with severe public health and economic impacts. While tick-borne diseases significantly impact human and animal health and agricultural production worldwide, as ectoparasites, ticks can also cause serious tissue injury, tick paralysis, or exsanguination from mass infestation. Experimental tick feeding is necessary to study tick-borne diseases and effectively test novel vaccines and therapeutics. Such studies raise concerns about on-host tick containment. Classically, tick containment cells for feeding on animals are rigid, lidded containers that are adhered to the host’s skin with adhesive or tape. They are bulky and easily damaged.

**Methods:**

Here, we describe the use of mesh packets, termed ‘teabags,’ containing 20 male and 5 female ticks each of both *Amblyomma americanum* and *Dermacentor variabilis* applied with surgical tape beneath harnesses on five dogs to allow tick feeding. Canine hematological and blood chemistry parameters were recorded before, during, and after tick feeding.

**Results:**

Successful feeding for 7 days was observed for both tick species (21–24/25 *A. americanum* and 3–14/25 *D. variabilis* per dog). Statistically significant shifts were detected in canine host hematological and blood chemistry parameters during tick feeding, indicating that infestation with even small numbers of ticks affects the systemic hematological and blood chemistry parameters.

**Conclusions:**

This new method is safe, humane, and effective and will improve the experimental design, containment, and safety of tick-feeding research across many host, parasite, and pathogen species.

**Graphical Abstract:**

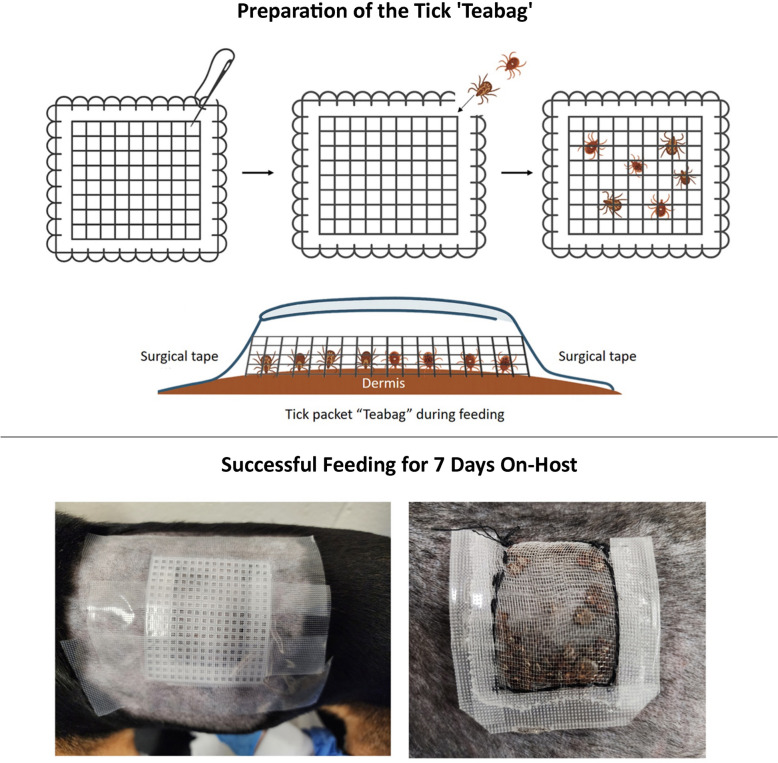

## Background

Hard ticks transmit a wide range of viral, bacterial, and protozoal pathogens that cause both acute and chronic infections, resulting in fatal or debilitating diseases in people, livestock, and companion animals [1, 2]. Viruses like Powassan, Bourbon, and Heartland are little studied, although they can cause potentially fatal illnesses; thus, treatment is purely supportive, and vaccines are mostly nonexistent [3–5]. In Europe, the tick-borne encephalitis virus causes debilitating or fatal infection and is commonly associated with coinfections with other tick-borne pathogens in humans and livestock [6]. The highly fatal Crimean-Congo hemorrhagic fever virus is transmitted by ticks throughout parts of Europe, Africa, and Asia, infecting humans and livestock [1, 7].

The tick-borne bacterial pathogen *Borrelia burgdorferi* causes acute and chronic Lyme disease with lifelong effects in several vertebrate hosts, including people [1]. Many tick-borne rickettsial pathogens belonging to the genera *Anaplasma, Ehrlichia*, and *Rickettsia* infect diverse vertebrate species and are often responsible for severe diseases with significant morbidity and mortality worldwide in humans, livestock, and companion animals [8]. For example, *Anaplasma marginale* causes severe hemolytic anemia in cattle globally and costs North American agricultural communities alone > 1 billion US dollars per year [9]. Similarly, *Rickettsia rickettsii*, the causative agent of Rocky Mountain spotted fever, infects humans and dogs throughout the Americas and causes high mortality rates ranging from 30 to 80% when not treated in the early stages of infection [10, 11]. Tick-borne protozoan parasites belong to the genera *Babesia* and *Theileria*, which infect livestock, increasing costs, reducing production, and causing high mortalities in herds [2, 12]. These pathogens vary widely in the tick species, vertebrate species, and regions of the world [1].

Pathogen transmission is not the only threat posed by ticks. Infestation with ticks alone can produce acute injury or disease; in extreme cases, high infestation loads can lead to death by exsanguination [13]. Studies have shown that infestation with as few as 60 female *Dermacentor andersoni* ticks on rabbits weighing 2–3 kg results in death by exsanguination after just over a week of feeding [13]. Some tick species are known to produce significant tissue-feeding lesions, while others secrete potent toxins, such as a neurotoxin of *Ixodes holocyclus* (the paralysis tick), which is reported to be fatal to adult dogs with infestation by a single tick [14]. In the USA, *Dermacentor andersoni* and *D. variabilis* single-tick infestations in humans have repeatedly caused tick paralysis, while *Amblyomma americanum* infestations in humans are linked to the development of α-gal syndrome, also known as red meat allergy [15–17].

Studies performed using ticks are limited partly because of biosecurity and containment requirements and because they are labor intensive [18]. Tick-feeding experiments are performed using many different host species, including dogs, cattle, sheep, and deer [19–29]. Such studies are necessary to advance the basic and translational research goals aimed at improving animal and human health [30–35]. Tick feeding results in the secretion of tick saliva proteins and metabolites having analgesic and immunomodulatory properties, which facilitate continuous tick feeding, largely preventing irritation and distress to the host [14, 36]. Ticks preferentially attach and feed in warm, covered areas of the body [37].

The canine host is an extremely important species for the study of tick infestations and tick-borne diseases [37, 38]. Dogs serve as an intermediary host, bringing ticks into proximity with their human owners, and they serve as reservoirs of infection for many tick-borne pathogens that pose high risks to humans. Increased stray dog populations with poor access to veterinary care in underserved regions are risk factors associated with a higher incidence of human cases of Rocky Mountain spotted fever (RMSF) [39]. Despite their great importnace, the canine host is the most challenging to use in tick-feeding experiments.

Tick-feeding experiments require tick containment during on-host feeding, that must allow ticks to access the dermis to attach and feed for long time periods, which can span from 1 to 4 weeks or more, depending on the tick's life stage and species. Thus, tick containment systems must remain in good condition for the entire duration of tick-feeding experiments and be either resistant to or protected from tampering by the host. Different host species bring different challenges. Livestock such as sheep and cattle can be stanchioned for a week or longer with supervised exercise time, whereas dogs cannot be physically restrained in this manner because of their highly active nature. Prior on-host containment methods for tick-feeding studies comprise a fabric sleeve (for stanchioned livestock) or a plastic capsule with a lid adhered to the animal by adhesive or tape [18]. These methods rely upon the host dermis as part of the containment [18, 40]. The capsules can be bulky, making it difficult to use harnesses over the top of a containment capsule to prevent pawing or rubbing on housing structures. The host's ability to tamper with the containment system is problematic when designing studies involving serious and deadly pathogens.

In this study, we tested a new tick-feeding containment method, referred to as the ‘teabag method,’ designed to minimize this risk by providing a secure, self-contained, and host-independent tick containment system. The teabag method uses a very small and thin tick containment chamber, and it is self-contained (not relying on the host's epidermis as part of the containment) for safe, effective, and easy on-host placement. The method employs a containment packet that can be prepared days or weeks in advance, transported, and placed on a host. This method is a major advancement in containment of tick feeding and in safety for medium and large animal species.

## Methods

### Animals and ticks

Five 14-month-old neutered male beagles were transferred from another study. Adult *Dermacentor variabilis* and *Amblyomma americanum* ticks were purchased from the Oklahoma State University tick-rearing laboratory and maintained in a chamber humidified by saturated potassium sulfate solution with a 14 h day/10 h night lighting cycle at 25 °C until being used in the study. Dogs were acclimated and trained to wear service dog harnesses (listed below) for over 1 month before tick placement.

### Installation of the teabag containment

**Fig. 1:**
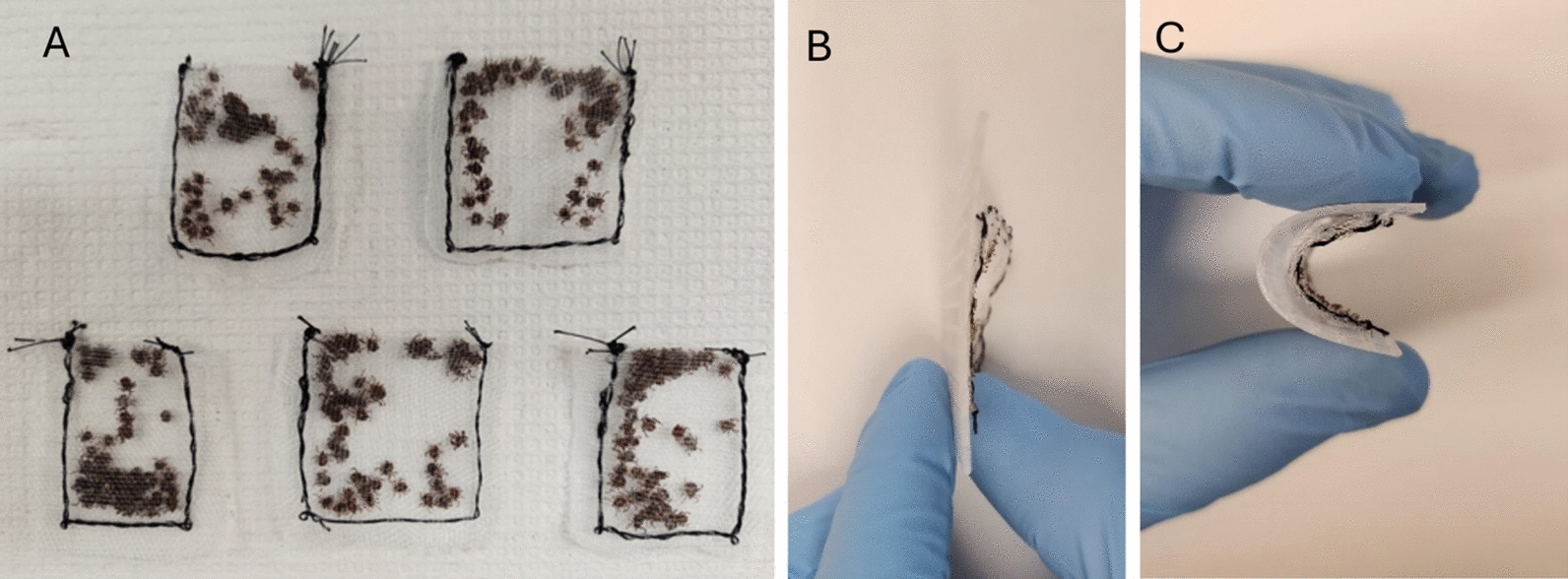
Teabag containment packet size, profile, and flexibility. **A** Tick containment ‘teabags’ containing 50 adult ticks in a 2 inch × 2 inch packet. **B** Profile of the tick containment packet with the embroidery canvas backing. **C** Flexibility of the tick containment packet with embroidery canvas backing. A single packet's profile, straight and flexed, is shown

Polyethylene bug netting (Meuallikit, Ultra Fine Garden Netting) was cut to size (~ 1.5 × 3 inches) and stitched using thread to form a pouch (the ‘teabag’), leaving one corner unstitched to insert the ticks. Using forceps, ticks were carefully inserted through the open corner of the teabag until all ticks had been transferred into the bags. We used 20 males and 5 females each of *Dermacentor variabilis* and *Amblyomma americanum* for the study. The teabags were sealed by stitching the open corners. Surgical tape was used to seal the teabag perimeter, reinforcing the stitched areas. The teabags were all 1.5–2 inches wide and long, forming square bags. Embroidery canvas (Caydo 6 Piece, 7 Count Plastic Mesh Canvas Sheets, Eye Mesh for Therian Masks, Acrylic Crafting Yarn, Knit Crochet Projects, and Make Aquarium Dividers [10.5 × 13.5 inches]) was cut to the desired size, which was slightly larger than that of the teabags (0.25 to 0.5-inch overhang on each side). Completed teabags and embroidery canvas covers had a narrow profile and were flexible (Fig. 1B, C).

Fur on the left side of each dog was clipped, and the skin was washed with dawn dish soap, dried thoroughly with a towel, and allowed to air dry. As soon as the skin was dry, the teabags containing both tick species were placed and directly covered by the embroidery canvas. Surgical tape (MED PRIDE 2 inch × 10 yard Transparent Surgical Tape 6 Pack with Medical Tape Case—Breathable Hypoallergenic Latex-Free Medical Adhesive Tape) was applied firmly to cover all four sides of the embroidery canvas, covering an inch of skin on each side. The embroidery canvas provided support and pressure for the teabag while remaining flexible and preventing the tape from directly contacting the teabag or ticks inside. Subsequently, a service dog harness (WINSEE Mesh Dog Harness with 10 Pet Patches for Small Service Dogs in Training, Breathable Tactical Molle Vest with Double Handles, Reflective Military Pet Vest for Walking Hiking) was placed, covering the entire packet and tape area on the dogs and was secured via a cotton cord to an appropriately sized Elizabethan collar. Dogs were returned to pens and housed individually during tick feeding. Dogs and containment were checked twice daily, and the tape was replaced as often as needed. Ticks were removed after 7 days of feeding by gently pulling on the teabags and simultaneously detaching individual ticks using forceps.

### Blood collection and monitoring

All dogs were monitored twice daily during tick feeding, while blood was collected 10 days prior to tick feeding and on days 3 and 7 post-tick bag attachments. The teabags were removed on day 7, and blood was collected on day 14 after ticks had been removed (Day 21 post tick placement). Whole blood was collected in vacutainer tubes containing EDTA or heparin (2 ml blood volume each). Complete blood count (CBC) analysis was performed with an IDEXX Procyte Dx hematology analyzer using EDTA-treated whole blood. Plasma was collected from heparin-treated blood samples by centrifuging the blood for 15 min at 1500 × g at 4 °C. All plasma samples were aliquoted and stored at – 20 °C. Blood chemistry was performed using the heparin plasma samples from all dogs; the samples were thawed at room temperature and vortexed, and 250 µl was dispensed into the IDEXX Catalyst One analyzer and tested with a Chem10 clip.

### Data analysis and statistics

Hematological and blood chemistry parameters were plotted in GraphPad Prism10 and evaluated using paired t-test statistics.

## Results

**Fig. 2:**
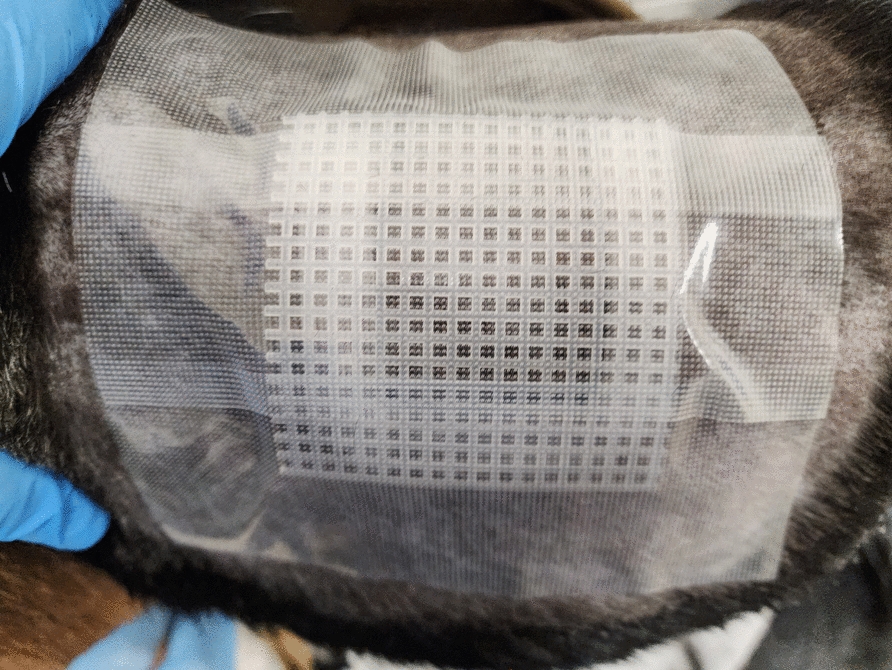
Placement of the ‘teabag.’ The teabag containment packet attached via surgical tape to the canine host for feeding. The embroidery canvas backer prevents direct contact between the tape and the tick-feeding packet and distributes pressure for even, firm contact across the teabag

**Fig. 3:**
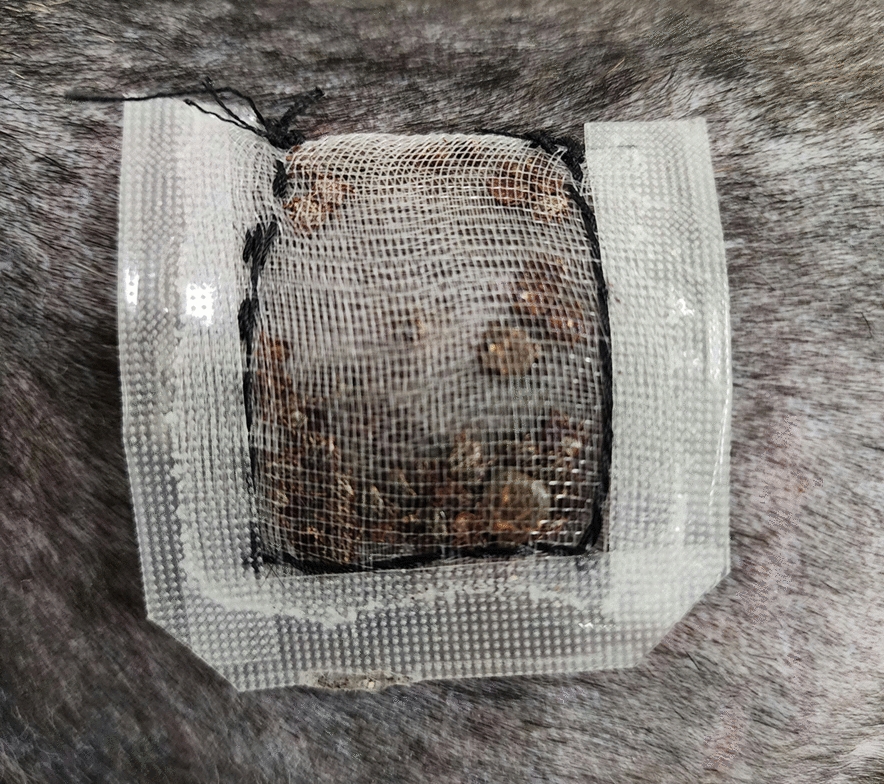
Tick-feeding efficiency. Partially engorged females and attached males are visible through the bags. The skin around the containment appeared healthy and was not irritated

The teabag method effectively contained ticks, prevented the host from tampering with the containment, and permitted tick feeding. The teabags, prepared as outlined in the methods section (Fig. 1), were first assessed in the laboratory setting before placement on the host (Fig. 2); ticks were effectively contained in the teabags. Assembly of the containment bags on the dogs was effective and caused no distress. No damage to the containment teabag was seen over 7 days of tick feeding on dogs. Ticks on the dogs attached to the dermis through the mesh. Attachment of ticks through the mesh secured the teabags in place on the host (Fig. 3). The flexibility of the mesh teabags and the activity level of the more active dogs allowed some of the smaller sized ticks that had securely attached to the dogs for feeding to be pulled through the containment teabag mesh. These ticks, however, remained within the surgical tape barrier on the host, which served as a secondary containment. During the twice-a-day checks, any ticks outside of the teabags were removed from the host with forceps, recorded as fed ticks, and returned to the laboratory. All ticks on the most active dog (dog ID HWJ) were removed on day 3 of tick feeding. Ticks that remained inside the teabag from dog HWJ were evaluated, and the containment bag was reattached. Upon checking on day 4, no ticks were feeding, so the ticks and the containment bag were removed from HWJ. All other dogs retained the teabags with ticks feeding within them for the entire 7 days of the study. At the completion of the study, all ticks were counted and recorded based on species, sex, and feeding status (Table 1).

**Table 1:** Tick-feeding success for the two hard ticks assessed

Tick-feeding success (fed ticks/total)				
	*Amblyommaamericanum*		*Dermacentorvariabilis*	
Dog	Male	Female	Male	Female
HWJ	9/20	1/5	0/20	2/5
GFH	18/20	4/5	11/20	3/5
GEH	20/20	4/5	8/20	4/5
IGH	18/20	4/5	10/20	4/5
FUH	17/20	4/5	1/20	2/5

### Dogs remained clinically healthy throughout the study

Physical examination showed that all dogs remained normal during the tick feeding, with no clinical signs, having unaltered behavior, normal temperature, and normal hydration status. The tick-feeding sites where the teabags with ticks were attached showed no tissue damage other than the normal visible tick-feeding lesions.

### Tick feeding caused changes in hematological and blood chemistry parameters

**Fig. 4:**
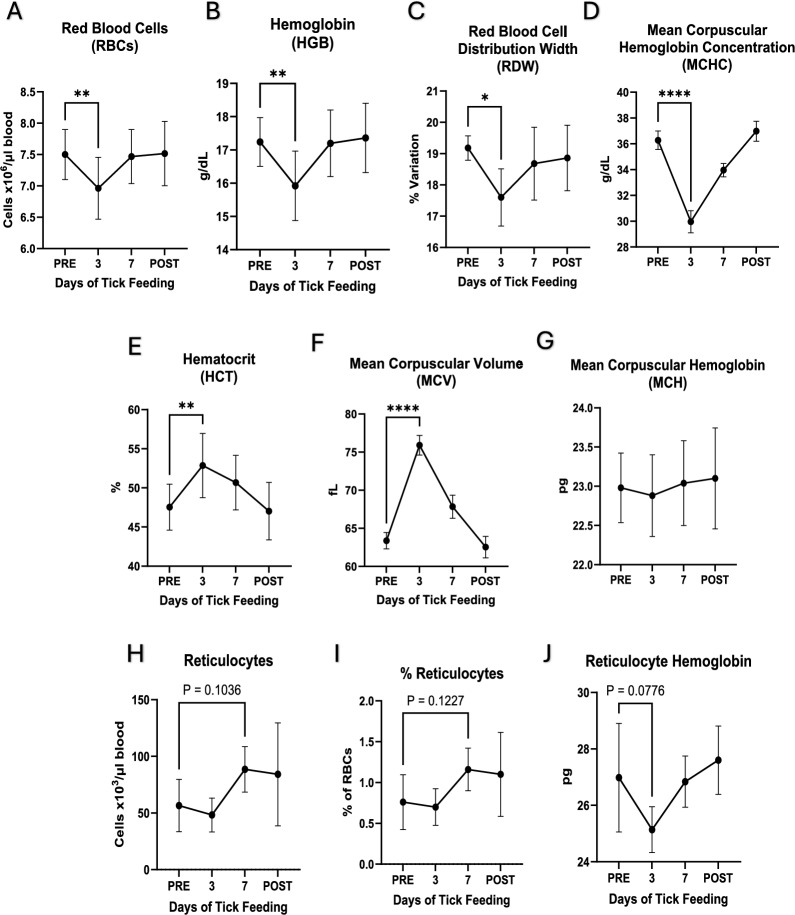
**Red blood cell parameters**. PRE stands for samples collected prior to tick placement andfeeding while POST refers to samples collected 14 days after tick removal, 3 and 7 refer to the numberof days after tick placement and active feeding. A) RBCs, B) HGB, C) RDW, D) MCHC, E) HCT, F) MCV,G) MCH, H) reticulocytes, 1) % reticulocytes, and J) reticulocyte. Graph generation and statisticalanalyses were performed in Graphpad Prism 10. Statistical analysis performed as paired t-test, * *p* ≤0.05, ** *p* ≤ 0.01, *** *p* ≤ 0.001, **** *p* ≤0.0001

In all dogs, several red blood cell parameters were significantly different (*p* ≤ 0.05) on day 3 of tick feeding compared with the pre-tick-feeding timepoint (PRE) (Fig. 4). These parameters included decreased red blood cell (RBC) count, hemoglobin (HGB) value, red blood cell distribution width (RDW), and mean corpuscular hemoglobin concentration (MCHC) (Fig. 4A–D). Both hematocrit (HCT) and mean corpuscular volume (MCV) were increased significantly on day 3 of tick feeding (Fig. 4E, F). Reticulocytes had an increased trend after 7 days of tick feeding, but there was no significant change (Fig. 4H, I). Similarly, no significant differences were observed in blood values 14 days after removing the ticks from the dogs.

**Fig. 5:**
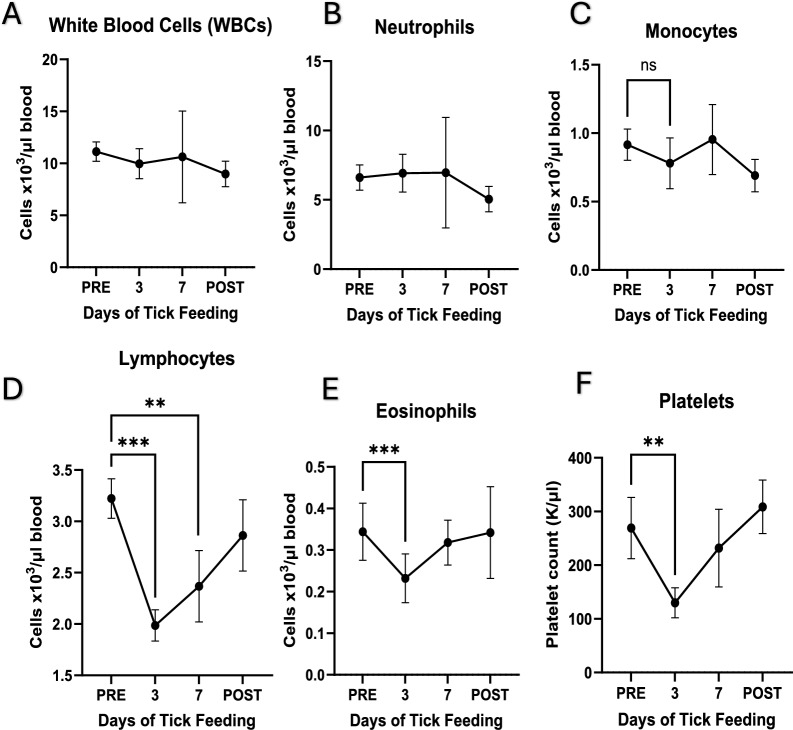
**White blood cell parameters**. PRE stands for samples collected prior to tick placement andfeeding while POST refers to samples collected 14 days after tick removal, 3 and 7 refer to the numberof days after tick placement and active feeding. A) WBCs, B) neutrophils, C) monocytes, D)lymphocytes, E) eosinophils, and F) platelets. Graph generation and statistical analyses performed inGraphpad Prism 10. Statistical analysis performed as paired t-test, * *p* ≤ 0.05, ** *p* ≤ 0.01, *** *p* ≤ 0.001

Total white blood cell counts were stable during the tick feeding, with neutrophil and monocyte counts remaining unchanged on day 7 post-tick feeding (Fig. 5A–C). Lymphocyte, eosinophil, and platelet counts were significantly decreased on day 3 of tick feeding, and lymphocyte counts remained decreased also on day 7 of tick feeding (Fig. 5D–F). The values returned to the pre-tick feeding state when assessed on day 14 post-tick removal.

**Fig. 6:**
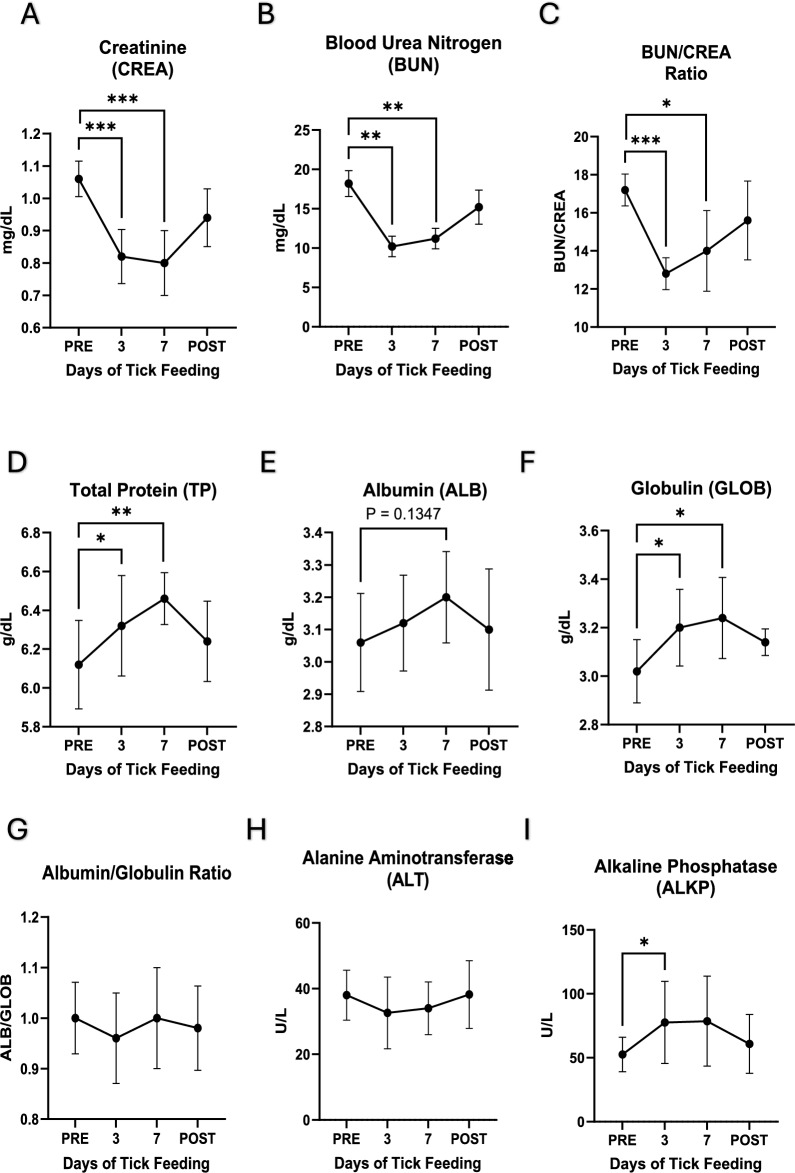
**Blood chemistry parameters**. PRE stands for samples collected prior to tick placement andfeeding while post refers to samples collected 14 days after tick removal, 3 and 7 refer to the number ofdays after tick placement and active feeding. A) CREA, B) BUN, C) BUN/CREA ratio, D) ,TP E) ALB, F)GLOB, G) ALB/GLOB ratio, H) ALT, and I) ALKP. Graph generation and statistical analyses performed inGraphpad Prism 10. Statistical analysis performed as paired t-test, * *p* ≤ 0.05, ** *p* ≤ 0.01, *** *p* ≤ 0.001,**** *p* ≤0.0001

Several blood chemistry parameters also changed significantly during tick feeding. Plasma creatinine (CREA), blood urea nitrogen (BUN), and BUN/CREA ratio were significantly decreased after day 3 and 7 of tick feeding, while total protein (TP) and globulin (GLOB) levels were significantly increased at the same timepoints (Fig. 6A–F). Alkaline phosphatase was increased significantly on day 3, while a similar increase was observed on day 7, but the *p* value for this timepoint was 0.0717 (Fig. 6I). When dog blood samples were assessed 2 weeks after tick removal, the parameters were found to be restored to pre-tick-feeding levels.

## Discussion

We presented a new method for containing ticks during on-host feeding experiments. The teabag method is safe and effective, allowing successful attachment and feeding on dogs. Efficacious tick-feeding methods are necessary to support research on developing therapeutics and vaccines against ticks and tick-borne diseases, further defining the biology of tick feeding and host immune responses. The teabag method is easier, safer, and more comfortable than previously described methods [18, 40]. The previously established methods require attaching tick cells to the skin as part of the containment, which irritate the skin and promote moisture buildup [18, 40]. Thus, the teabag method will be valuable for improving biosafety in studies with more dangerous pathogens, such as *Rickettsia rickettsii*. The method was successfully tested with two tick genera. It is non-irritating to the host and produces no discomfort to the host beyond that of natural tick infestations, which are common with heavier loads of infestation than those used experimentally.

While the canine host is one of the most challenging models for tick-feeding studies, it is also one of the most important model organisms for research on ticks and tick-borne diseases as dogs are reservoirs for several zoonotic tick-borne pathogens and host tick infestations and live in direct contact with human families worldwide [37, 38]. In the North American Southwest and Central America, family dogs are important reservoirs of infection for *R. rickettsii* and tend to be infested with *Rhipicephalus sanguineus* (commonly known as the brown dog tick) [41]. This tick is a vector for the Rocky Mountain spotted fever (RMSF) agent, *R. rickettsii*, and resides in and around the houses of infested dogs [41]. The tick inevitably feeds on humans within a household and transmits *R. rickettsii*, causing RMSF, with high mortality rates ranging from 30–80%, particularly in children [10, 11]. In Arizona, a large canine populations with limited access to veterinary care correlate strongly with human cases of RMSF [39]. This is just one example of why tick-feeding studies on dogs are especially important for human preventive medicine and for developing vaccines and therapeutics in support of the One-Health objective of treating human and animal diseases together.

The teabag method involved using polyethylene bug netting as the mesh for the teabag. The mesh size used is suitable for adult ticks; however, it is too large to contain nymphs and larvae. The method can easily be adjusted to a user’s need, e.g., by using a finer mesh size for studies involving smaller size adult ticks and early tick life stages, such as for nymph and larval infestation studies . Also, due to the flexibility of the polyethylene mesh used in this study, smaller ticks attached firmly to the host can be pulled through the mesh by the host's movements (in this case, highly active dogs) or by staff performing containment checks. These ticks are all retained within the secondary tape barrier until removal; however, for other studies, a sturdier material (such as steel mesh) may be selected for the mesh.

In the current study, we also investigated the impact of tick feeding on the hematological and blood chemistry parameters in dogs. The hematological changes observed in the current study did not cause the parameters to fall outside clinically normal ranges, but they were statistically significant. The data demonstrate that tick feeding has a systemic impact on the host. We observed that red blood cells were heavily impacted by tick feeding by day 3 after tick placement but usually recovered by day 7, indicating that the canine host response compensated for the impact of tick feeding, at least during the short duration of assessment. Natural infestations last longer and incur much higher infestation loads, amounting to hundreds of ticks per host, and they can lead to fatal blood loss [13].

Although the values remained clinically normal, the changes in RBCs indicate a regenerative state of the RBC population. Mild decreases in RBC count indicate blood cell loss, while increases in the hematocrit are a result of the larger individual RBC size. The decreases in MCHC along with the increase in MCV indicate a shift to less mature RBCs being released as the primary population of RBCs in circulation. The lack of a true reticulocytosis demonstrates the mild nature of this regenerative blood loss, as the analyzer did not characterize the less mature RBCs as true reticulocytes. The decrease in lymphocytes and eosinophils observed during tick feeding may be due to the cells leaving the circulation and entering the tick-feeding site. It is also possible that lymphocytes migrated to secondary lymphoid organs to process tick salivary proteins, some of which are known to be highly antigenic [36]. The sharp decline in platelets by day 3 could indicate the failure of the body to stop the blood loss due to tick feeding or increased vascular leakage secondary to tick salivary effects. The recovery of platelet values by day 7 clearly demonstrates compensation by the canine host to the effects of the infestation. The observed changes in blood chemistry were minor and remained within clinically normal ranges, causing no severe changes in animal health status. Blood chemistry parameters, including decreased BUN, CREA, and BUN/CREA ratio and increases in TP, GLOB, and ALKP, indicate that there may be a very mild and transient inflammatory effect on the liver. Under specific physiological or clinical conditions, increases in hematocrit (HCT) and albumin (ALB) can occur alongside reductions in urea (BUN) and creatinine (CREA) in dogs. While dehydration is a common cause of increased hematocrit (HCT) and albumin (ALB), the decrease in RBC count and stable WBC counts indicate that these data reflect systemic compensation and responses to infestation, likely affecting multiple organ systems rather than dehydration. While these data are interesting, additional, more detailed investigations are warranted to better understand the impacts of tick feeding and the host response.

## Conclusions

In summary, we report the novel teabag method for tick feeding, which will greatly improve tick-feeding experimental design, particularly when working with highly active vertebrates, such as dogs. This method is less stressful for the animals, thereby improving animal welfare and facilitating future studies of ticks and tick-borne diseases that impact humans, agriculture, and companion animals. This study also demonstrates the broad effects of small tick infestation loads on the systemic hematological and blood chemistry profiles of the canine host. The observed changes were significant but within normal ranges.

## Data Availability

This is to confirm that all the data generated in this study are included as part of the manuscript files. There are no additional data that are not part of the manuscript.
